# A Review of the Mechanism of Vascular Endothelial Injury in Immunoglobulin A Vasculitis

**DOI:** 10.3389/fphys.2022.833954

**Published:** 2022-03-16

**Authors:** Shanshan Xu, Shanshan Han, Yanlin Dai, Long Wang, Xia Zhang, Ying Ding

**Affiliations:** ^1^Pediatric Kidney Disease Center, Henan University of Traditional Chinese Medicine, Zhengzhou, China; ^2^Pediatric Kidney Disease Center, The First Affiliated Hospital of Henan University of Traditional Chinese Medicine, Zhengzhou, China

**Keywords:** system of complement, vascular endothelial injury, metabolomics markers, gene polymorphisms, immunoglobulin A vasculitis

## Abstract

Immunoglobulin A (IgA) vasculitis (IgAV), also known as Henoch-Schönlein purpura, is the most common form of childhood vasculitis. It is characterized by cutaneous hemorrhage, resulting from red blood cell leakage into the skin or mucosae, possibly caused by damage to small blood vessels. These acute symptoms usually disappear without treatment. Endothelial cells are distributed on the inner surfaces of blood vessels and lymphatic vessels, and have important functions in metabolism and endocrine function, as well as being the primary targets of external stimuli and endogenous immune activity. Injury to endothelial cells is a feature of IgA vasculitis. Endothelial cell damage may be related to the deposition of immune complexes, the activation of complement, inflammatory factors, and chemokines, oxidative stress, hemodynamics, and coagulation factors. Both epigenetic mechanisms and genetic diversity provide a genetic background for endothelial cell injury. Here, research on the role of endothelial cells in allergic IgA vasculitis is reviewed.

## Introduction

Immunoglobulin A (IgA) vasculitis (IgAV) is a systemic disease typified by leukocyte burst vasculitis, involving the deposition of capillaries and IgA immune complexes (Pillebout and Sunderkötter, 2021). Over 90% of IgAV patients are below the age of 10 (Gardner-Medwin et al., 2002; Yang et al., 2005; Leung et al., 2020). Epidemiological studies have shown that the incidence of IgAV is higher in Asians than in Caucasians and Africans (Gardner-Medwin et al., 2002). Renal injury, known as IgA vasculitis with nephritis (IgAVN), is a major manifestation in IgAV, with potentially fatal outcomes. During the first 4–6 weeks of IgAV onset, about 40% of children with IgAV may develop IgAVN (Saulsbury, 2010), and persistent purpura, severe abdominal symptoms, and older age are three risk factors for IgAVN (Buscatti et al., 2018). It is important to consider IgAV in clinical diagnosis, differential diagnosis, and treatment. Understanding the pathogenetic mechanism of IgAV is necessary for the provision of suitable treatment and medication, and this involves investigation of the association between vascular endothelial injury and IgAV.

Endothelial cells (ECs) are flat cells that form a highly differentiated monolayer on the inner surfaces of blood and lymphatic vessels. ECs have vital metabolic and endocrine functions in the human body. They are responsible for maintaining vascular permeability, stability of circulation, and anticoagulation, and are also the primary targets of attack by external stimuli and immune complexes (Yang et al., 2002; Cardinal et al., 2018). Injury to ECs is the first step in the development of a variety of vascular conditions, such as atherosclerosis (Kim et al., 2021), diabetic nephropathy (Mahdy et al., 2010), and hypertension (Li et al., 2021). Recent evidence has linked EC injury to the pathogenesis of IgAV, together with the development of proteinuria. This can lead to glomerular sclerosis, renal interstitial fibrosis, and damaged renal function. Matrix deposition is a pathological outcome and contributes to the formation of vascular lesions; this includes the deposition of immune complexes, metabolites, and enzymes such as oxidases and proteases, and is closely related to immune vascular damage. Matrix deposition is coordinated by the complement system, inflammation, the immune response, and metabolic abnormalities, in association with genetic polymorphism, and leads to the replacement of normal tissue. This replacement leads to abnormal cellular respiration and renal vascular hypoxia, with an increase in reactive acidic products, promoting the contraction of vascular endothelial cells and the widening of the inter-cellular spaces, leading to hematuria and renal fibrosis in a vicious circle that eventually results in kidney failure. In this review, we discuss EC injury in terms of complement activation, the formation of IgA1 immune complexes, chemotactic and inflammatory cytokines (Heineke et al., 2017), coagulation factors, epigenetics, and genetic polymorphisms, amongst other factors, in the pathogenesis of IgAV.

## Immunoglobulin A-Containing Immune Complexes

In IgAV, galactose-deficient IgA1 (Gd-IgA1) can be detected not only in the serum but also in the skin and kidney tissue (Neufeld et al., 2019; Oni and Sampath, 2019; Zhang et al., 2020), and IgA1-containing immune complexes, especially IgA1 accumulation in vessel walls, promote the development of IgAV. A multi-hit hypothesis is generally considered to illustrate the role of Gd-IgA1 in the pathogenesis of IgAV. IgA is a major class of immunoglobulins present in mucosal secretions where they are closely involved with mucosal immunity. There are two IgA subclasses, IgA1 and IgA2, with approximately 90% of circulating IgA monomers belonging to IgA1. The hinge region of the IgA1 molecule contains three to six O-glycosylation sites allowing the addition of Gal-GalNAc disaccharides. These glycosylated Gd-IgA1 proteins auto-aggregate or bind to IgG molecules that recognize galactose-deficient IgA. These immunoglobulin complexes may be too large to access the space of Disse in the liver and are, therefore, able to avoid coming into contact with hepatic receptors and can thus avoid degradation by hepatic cells. The IgA1 complexes thus accumulate in the circulation where they bind and activate FcαR1 transmembrane receptors on ECs, forming a soluble IgA1-sCD89 complex (van Zandbergen et al., 1999). This induces a widespread pro-inflammatory reaction involving the recruitment of neutrophils, activation of downstream signaling pathways, the release of neutrophil extracellular traps (NETs) resulting in the induction of NETosis and elevation of the levels of reactive oxygen species (ROS). Antibody-mediated cytotoxicity may also occur, together with cytokine and chemokine secretion, leading to EC injury (Aleyd et al., 2014; Heineke et al., 2017; Takeuchi et al., 2021). Furthermore, the activation of FcaR1s triggers the release of leukotriene B4 (LTB4), which activates and attracts neutrophil migration, forming a feedback loop (van der Steen et al., 2009). The pro-inflammatory cytokine, tumor necrosis factor-alpha (TNF-α), which is released by neutrophils, can activate ECs, inducing them to expose the hidden β2-glycoprotein I antigen (β2GP I) (Kim et al., 2021). Recognition of anti-endothelial cell antibodies (AECA) in combination with β2GP I activates the MEK/REK signaling pathway, along with the release of IL-8 and chemokines that attract polymorphonuclear leukocytes and monocytes (Yang et al., 2006, 2012). Pathogens, such as bacteria or viruses, induce similar IgA activities that are able to crosslink with ECs to propagate downstream signals. IgA1 complexes also stimulate mesangial cells through the transferrin receptor CD71 to trigger both proliferation and matrix production, leading to the release of angiotensin II, nitric oxide synthase, and cytokines, which appear to play key roles as direct or indirect effectors of EC damage by triggering acute and chronic inflammatory reactions (Chen et al., 1994; Novak et al., 2012).

**FIGURE 1:**
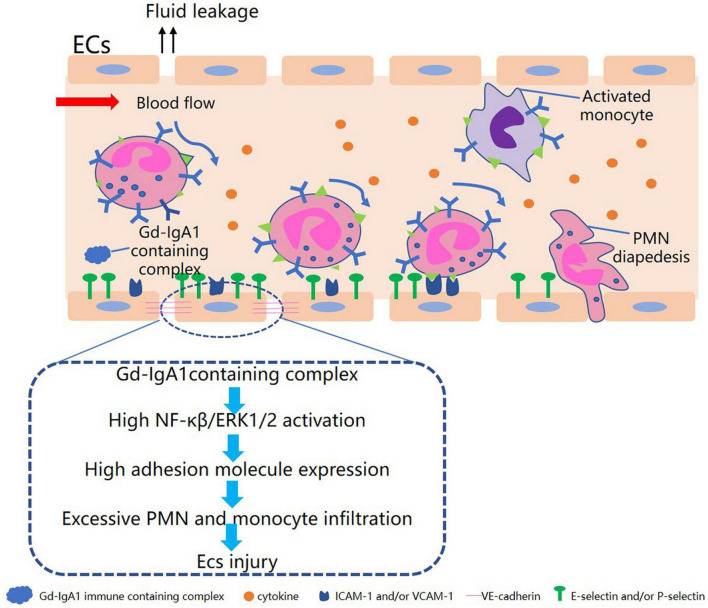
Diagram of Gd-IgA1-containing complex in relation to endothelial cell injury in IgAV.

It has been found that the sera of patients with active IgAV can induce the production of the chemokines CCL5, CXCL16, and CXCL1, as well as promote migration in dermal microvascular ECs and the human HL-60 leukemic and THP-1 monocytic cell lines (Chen et al., 2011a). It has also been found that patients’ sera promoted the translocation of nuclear factor-κB (NF-κB) p65 to the nucleus and stimulated phosphorylation of the extracellular signal-regulated kinase ERK1/2 protein. These findings indicate that sera from patients with active IgAV may damage ECs and stimulate chemokine secretion through the NF-κB and ERK1/2 pathways (Figure 1). Yuan et al. (2014) observed upregulation of the pro-apoptotic protein Bax and downregulation of the anti-apoptotic protein Bcl-2 in ECs cultured with IgA1 isolated from IgAV patients. This suggests that IgA1 can induce EC apoptosis, which may be linked to the vascular endothelial injury seen in IgAV. This IgA1-induced apoptosis of ECs may occur through the activation of apoptotic cell protease activator-1 and pro-proteogen-9, forming apoptotic bodies and reducing the downstream effectors, cysteine proteases 3, 6, and 7 (Steinberg et al., 2007). In summary, IgA1-containing immune complexes can induce inflammatory reactions by activating inflammatory signaling pathways and recruiting neutrophils, together with regulating the expression of apoptosis-related proteins, all of which could ultimately result in EC injury and promote the development of IgAV.

## The Complement System

The complement system consists of soluble proteins, membrane-binding proteins, and complement receptors. It forms a part of the human immune system, connecting the innate and acquired immune systems. When activated, it counteracts external threats, such as invading pathogens, and internal problems, such as fragmented material, to maintain the stability of the microenvironment. Deposits of complement components, including mannose-binding lectin (MBL), MBL-associated serine protease (MASP)-1, C3a, C5, and C5b-9, occur in both the skin and kidney in IgAV patients, indicative of the role of complement activation in IgAV (Endo et al., 2000; Hisano et al., 2005; Zhang Q. et al., 2019). The components of the complement cascade are present in inactive forms in the circulation, and may be activated by three pathways, the classical, lectin, and alternative pathways. Several studies have shown that IgA activates complement via the alternative and lectin pathways (Endo et al., 2000; Yang et al., 2015). Complement component C3 and the membrane attack complex (MAC) function in the final stages of all three pathways to produce components C3a and C5a, which act as chemotactic factors that attract neutrophils and induce the release of mediators to amplify inflammatory reactions. C3a and C5a levels are thus related to disease activity and can be used as sensitive indicators for both EC injury and IgAV monitoring. C3a and C5a act by promoting the complexation of p44/p42 kinase with G protein and specific factors, such as C3aR, that are expressed on ECs (Norgauer et al., 1993). Endothelial cells can themselves synthesize the proteins and regulators required for the complement cascade (Laufer et al., 2001), and activation of the complement system in the vascular wall can promote the secretion of cytokines and chemokines to enhance inflammation, leukocyte infiltration, and the immune response.

It has been found that stimulation of human umbilical cord endothelial cells (HUVECs) with C3a or C5a resulted in elevated mRNA expression and secretion of IL-1, IL-8, and RANTES by activated T cells (Monsinjon et al., 2003). IL-1 and IL-8 promote neutrophil migration to the sites of inflammation. Yang et al. (2015) found that C3a and C5a can up-regulate the production of monocyte chemoattractant protein 1 (MCP-1), endothelin 1 (ET-1), and intercellular adhesion molecule 1 (ICAM-1) by ECs. E-selectin and ICAM-1 are cell adhesion molecules expressed on the EC surface, and recruit neutrophils to inflammatory sites (Silva et al., 2002; Reichel et al., 2009). MAC, including lytic MAC and sub-lytic MAC, is produced in the common terminal pathway of the complement cascade, and may also be associated with EC damage. Lytic MAC can form channels in the cell membrane, thus disrupting membrane integrity and leading to cell osmotic dissolution. Sub-lytic MAC inserts into membranes increasing their permeability and causing both an influx of Ca^2+^ and a rapid reduction in the potential of the inner mitochondrial membrane, in both cases leading to cell death (Papadimitriou et al., 1991). In addition, MAC can also activate the p38 MAPK and EPK1 signaling pathways, promoting cytokine and chemokine production and subsequent immune cell infiltration. C5b-9 also can induce cell death by means of Bid cleavage and caspase activation (Ziporen et al., 2009). Plasma S protein binds to C5b-9 to form the water-soluble SC5b-9 complex, which is unable to bind to the cell membrane and is thus trapped within the cell. SC5b-9 has been found to promote upregulation of osteoprotegerin in ECs, which may aggravate inflammation (Corallini et al., 2009). MAC also promotes the activation of CDK4 and CDK2, leading to increased cellular proliferation through enhancement of the G1/S transition (Tegla et al., 2011). C5b-9 has also been found to block apoptosis through Bad phosphorylation and preventing FLIP activation (Tegla et al., 2011).

## Inflammatory Cytokines and Chemotactic Factors

IgAV is considered an inflammatory disease characterized by leukocytoclastic vasculitis. It has been reported that serum TNF-α, IL-1β, and IL-6 levels are elevated in patients with IgAV compared with healthy subjects (Pillebout et al., 2017), indicating the potential involvement of cytokines in the vascular damage seen in IgAV. IgA-containing complexes are potent inducers of neutrophil infiltration through the activation of the NF-κB, ERK1/2, and MEK/REK pathways, resulting in the propagation of downstream signaling in IgAV (Yang et al., 2006, 2012; Chen et al., 2011a). Cytokines such as IL-1β, IL-6, and TNF-α are known to stimulate chemokine release by ECs, resulting in the shedding of the EC glycocalyx and elevated expression of cell adhesion molecules, enhancing the attachment of the cells to the vascular wall (Yuan et al., 2018). Specifically, TNF-α can increase ROS production in vascular ECs and promote the short-term expression of ICAM-1 through activation of the ERK1/2 and p38 MAPK signaling pathways, leading to EC apoptosis (Izawa-Ishizawa et al., 2012). On the other hand, TNF-α enhances the binding of IgA AECA to ECs promoting the release of IL-8, ultimately leading to vascular damage (Yang et al., 2006). The balance between anticoagulation and pro-coagulation on ECs is affected by IF actor, which is produced with TNF-α to stimulate ECs, inducing thrombin production and fibrin deposition (Napoleone et al., 2002; Ge et al., 2014). Tumor necrosis factor-like weak inducer of apoptosis (TWEAK) is a newly described member of the TNF superfamily that can activate the non-canonical pathway of NFκB by phosphorylation of the κB-α inhibitor IκB-α and processing of p100, raising the levels of both adhesion molecules and chemokines in vein ECs (Saitoh et al., 2003). Transforming growth factor-β (TGF-β) also promotes the IgA isotype switch in B cells, thus contributing to the formation of antibodies that may cross-react with cardiolipin to produce vessel-damaging complexes (Yang et al., 2000).

The chemokine MCP-1 can bind to membrane G protein-coupled receptors to induce the PLC-IP3-mediated release of intracellular calcium, which can activate the NF-κβ signaling pathway, leading to the migration of monocytes to areas of inflammation (Melgarejo et al., 2009; Wang et al., 2018). It also stimulates mast cells to produce LTB4 and platelet-activating factor (PAF), both of which promote neutrophil migration and infiltration (Silva et al., 2002; Reichel et al., 2009). The attachment of neutrophils to inflamed tissues requires both the action of chemokines and adhesion molecules on ECs, such as ICAM1 and E-selectin. Proteases and metalloenzymes are secreted by both leukocytes and ECs and can cause damage to the EC glycocalyx. Neutrophils and monocytes secrete the metalloproteinases MMP-2 and MMP-9, which not only destroy the integrity of the basement membrane by degrading type VII collagen (Kobayashi et al., 1998; Shin et al., 2011), but also activate α-actin, stimulate extracellular matrix formation, and increase cytokine secretion (Danilewicz and Wagrowska-Danilewicz, 2010). Toll-like receptors (TLRs) are transmembrane glycoproteins expressed by immune cells such as monocytes and macrophages, and trigger a cascade of intracellular signals when activated by pathogen-associated molecular patterns (PAMPs), leading to the activation of NF-κB and interferon regulatory factor (IRF) (Donadio et al., 2014), both of which are closely associated with the immune and inflammatory reactions.

## Hemodynamics

The renin–angiotensin system (RAS) regulates sodium homeostasis, blood pressure, and inflammation. It also modulates vascular tone and possibly vascular structure either directly or through interaction with factors such as endothelin and nitric oxide (NO), amongst others. A recent paper by Özkaya et al. (2006) indicated that the role of the RAS in IgAV pathogenesis should not be overlooked. A major component of the RAS, angiotensin II (Ang II), has been linked to both endothelial dysfunction and vascular inflammation, and is closely associated with Ang II-mediated ROS production and the activation of redox-dependent signaling cascades. Ang II promotion of ROS generation is also linked to NF-κB and NADP oxidase activation (Montezano et al., 2014; Zhang H. et al., 2019). ROS, in turn, triggers NF-κB expression, leading to the vascular inflammatory response through the action of IL-8, TNF-α, and TGF-β. Thus, increased ROS production induces a positive feedback loop, resulting in vascular injury. On the other hand, the binding of Ang II to its receptor impairs NO synthesis and increases endothelin secretion by reducing eNOS activity and promoting the uncoupling of NOS, ultimately leading to dysfunctional vasoconstriction and consequent damage to ECs (Montezano et al., 2014). The RAS can also induce EC apoptosis by increasing the concentration of cytochrome c in the cytosol and the modulation of apoptosis-related proteins, including the upregulation of apoptosis promoters (Bax, Bad, and caspases 3 and 9) and downregulation of the anti-apoptotic protein Bcl-2 (Chang et al., 2016) in the mitochondrial pathway. It is known that the mitochondria play major roles in the induction of apoptosis. Hitom et al. (2010) found that Ang II reduced the expression of MMP-9 in ECs, leading to mitochondrial dysfunction by increasing the levels of cytosolic cytochrome c and activating the caspase3/9 cascade. ROS, in turn, damages the mitochondria and reduces the expression of MMP. A previous study has also shown that lysosomal cathepsins are involved in apoptosis through crosstalk with the mitochondria (Chang et al., 2016). Ang II modulates the expression of VEGF and its receptors and induces monocyte proliferation and infiltration in ECs (Buyan et al., 1998). Ephrin is a newly described member of the RAS system. Ephrin binds to the receptor PRR, inducing a conformational change and mediating EC apoptosis through activation of the ERK1/2 signaling pathway. In view of the important role of RAS activation in vascular injury, angiotensin-converting enzyme inhibitor (ACEI) and angiotensin II receptor antagonist (ARB) are used to alleviate renal vascular damage in patients with IgAVN and delay the progression of the disease.

## Oxidative Stress

Oxidative stress caused by the disruption of the balance between cellular oxidants and antioxidants is known to result in cell damage. Oxidative stress resulting from the action of free radicals and ROS is recognized as the main cause of EC injury, resulting in reduced nitric oxide and increased ROS production. There are many routes for ROS production in the cell. Besides the mitochondrial pathway, ROS is also produced by the catalytic process of cyclooxygenases, lipoxygenases, xanthine oxidase, or other nicotinamide adenine dinucleotide phosphate (NADPH) oxidase subtypes. NADPH is the main source of ROS in the vascular system and is activated by Ang II, thereby increasing the release of ROS (Knock, 2019). Ece et al. (2007) have shown that myeloperoxidase (MPO) levels are elevated in the plasma of IgAV patients. MPO is secreted by activated neutrophils and ECs and reacts with hydrogen peroxide and chloride to form highly reactive toxic compounds such as hypochlorous acid (HOCL). HOCL is capable of amplifying oxidative stress, resulting in further damage to ECs. In addition to MPO, the key proteins of NETosis include NE and peptidylarginine deaminase IV (PAD4). ROS can induce the transcription and translation of PAD4, leading to its entry into the nucleus, where it citrullinates the histones, altering the overall charge on chromatin and promoting its disassembly. This leads to the dissolution of the nuclear and granular membranes, allowing antibacterial proteins in the cytoplasm to attach to the depolymerized chromatin to form a network structure that is subsequently released to the outside of the cell (Arneth and Arneth, 2021; Takeuchi et al., 2021). Both prostaglandin E and malondialdehyde levels are known to be elevated in IgAV, indicating activation of the cyclooxygenase pathway that induces ROS (Buyan et al., 1998).

Elevated ROS is not only directly cytotoxic but also affects mitochondrial respiration, leading to the peroxidation of the lipid components of cell membranes and causing severe damage to the membranes (Singh et al., 2020). Elevated levels of malondialdehyde, a product of membrane lipid peroxidation, have been reported in the plasma of IgAV patients, indicating ROS-mediated damage to ECs (Chen et al., 2011b). A previous study has shown that ROS also acts as a signal transducer between cells and can mediate the activation of NF-κB and Ras signaling pathways, stimulating the release of inflammatory factors (Pourova et al., 2010). ROS is also a potent inducer of apoptosis in ECs through alterations of the mitochondrial membrane potential and initiation of the caspase cascade (Pourova et al., 2010). Besides lipid peroxidation and apoptosis, excessive ROS also results in cellular injury caused by DNA damage and protein denaturation and dysfunction. ROS promotes the decoupling reaction of endothelial NO enzymes, inhibiting the production of NO by ECs, and also combines with NO to generate peroxynitrite, a strong oxidant, which damages EC DNA (Moncada et al., 1991). Evidence has shown that ROS activates neutrophil elastase, a neutrophil-secreted protease that, if not appropriately controlled, can result in severe damage to the extracellular matrix (Shah, 1989). Moreover, ROS also acts as a mediator of inflammation, damaging ECs through leukocyte infiltration and adhesion (Kouka et al., 2017). In summary, a variety of pathways lead to increases in ROS, which damages ECs through lipid peroxidation, DNA damage, and the promotion of apoptosis and signal transduction, resulting in increasing recognition of the importance of preventing oxidative stress to delay the progression of IgAV.

## Coagulation Factors

There are elevated levels of serum D−dimer, thrombomodulin, and PAF in patients with acute-phase IgAV. It has been found that anti-coagulation therapy can reduce the development of kidney damage, indicating the close relationship between coagulation/fibrinolysis disorders and IgAV pathogenesis. There are a number of reasons for the damage to ECs, including the presence of collagen fibers that allow attachment of blood platelets and coagulation factors that, as a result of activation, form clots, aggravating the EC dysfunction. Activated platelets recruit circulating leukocytes through interactions between platelet P-selectins and the P-selectin glycoprotein ligand-1, leading to the secretion of inflammatory factors such as TNF-α, MCP-1, and IL-8 (Elstad et al., 1995; Weyrich et al., 1995; Wang et al., 2005). P-selectin promotes the interaction between activated platelets and ECs, resulting the deposition of the platelet-derived pro-inflammatory factor RANTES on the blood vessel walls, thus aggravating EC damage (Schober et al., 2002; Butler et al., 2007). Activated platelets and P-selectin thus form a circular feedback pathway. PAF mediates the deposition of complement components and immune complexes in the endothelium and mesangium, leading to glomerular cell activation and inflammatory cell infiltration. The specific binding of thrombin to the thrombin receptor on the EC surface promotes the expression of the extracellular matrix-degrading enzyme, plasminogen activator. In ECs, resulting in the degradation of fibronectin in the matrix and the consequent detachment of ECs from the matrix (Noiri et al., 1995). This detachment not only enhances the coagulation process but also aggravates renal injury. In addition to activating platelets, thrombin has been reported to upregulate ICAM-1 expression in ECs, which, in turn, recruits neutrophils to the inflamed tissue. There are also receptors on the EC surface that bind specifically to avβ3 integrin and fibrin heparin-binding sites (Francis et al., 1993). Once fibrin forms near ECs, it will bind specifically to the cells, affecting both their morphology and function. Fibrin can not only induce changes in the EC phenotype but also destroys the structure of the EC monolayer. ECs spread spontaneously to form vascular-like structures on fibrin temporary matrices and upregulate the expression of ICAM-1 to mediate the infiltration of glomerular inflammatory cells (Takei et al., 1995). The plasminogen activator inhibitor reduces the hydrolysis of fibrin and the degradation of the extracellular matrix by blocking plasminogen activator activation (Shin et al., 2005). Activated platelets, thrombin, and fibrin play different roles in the EC injury process, leading to further coagulation disorders. Therefore, early intervention to prevent EC injury is essential.

## Genetic Polymorphism

There is considerable geographical and ethnic variation in the prevalence of IgAV, with higher rates seen in Asians than in Caucasians, and relatively low levels observed in the black population (Davin and Coppo, 2014; Pillebout and Sunderkötter, 2021). This suggests a strong influence of genetics in the development of IgAV. In support of this hypothesis, there is also an increased risk of IgAV in the first-degree relatives of IgAV patients, together with observations of familial aggregation (Zhang et al., 2008; Chen et al., 2012). Numerous investigations of candidate genes using high-throughput techniques have been carried out in IgAV patients. The studies found that genetic diversity in leukocyte antigens, inflammatory factors, chemokines, and the RAS system were related to both IgAV susceptibility and the development of IgAV-associated kidney disease in IgAV, as well as being involved in EC damage in IgAV. The HLA gene encodes the most polymorphic of human proteins, namely, the class I and II antigen-presenting molecules (Klein and Sato, 2000). HLA has been found to be a major genetic factor contributing to many inflammatory and immune disorders. HLA-A*2, HLA-A*11, and HLA-A*26 in the HLA class I regions have been related to the onset of IgAV, and HLA-B*44 and HLA-B*58 have been associated with kidney damage in IgAV patients (Peru et al., 2008; Ren et al., 2012). In addition to the HLA region encoding antigen-presenting proteins, genes located in the HLA class III region also encode complement proteins, which mediate immune responses and damage ECs. HSP70, encoded by HSPA2, is implicated in the formation of the extracellular matrix, while HSPA2 1267 G/A is related to inflammatory reactions (Pablos et al., 1995; Vargas-Alarcon et al., 2002). The inflammatory factor IL-6 is involved in both innate and acquired immunity and mediates inflammation by binding to specific receptors. The IL-6 promoter rs1800795-174 (G/C), IL-6R rs2228145 (A/C), and IL-6ST rs2228044 (C/G) are involved in EC injury (Palomino-Morales et al., 2009; Rodriguez-Rodriguez et al., 2011; Hirahara et al., 2016). The binding of leukocytes to the endothelium requires the mediation of adhesion molecules. Expression of the SELP-825 and SELP-2123 genes encoding P-selectin in IgAV patients has been shown to be increased, and the genotype of SELP-2123 GG and the SELP-2123 allele have been related to IgAV susceptibility (Li and Liu, 2011; Lopez-Mejias et al., 2018). The RAS modulates vascular tone and possibly also structure through various factors including NO and endothelin. Ang II is a vasoconstrictor associated with inflammation, and the Ang rs4762 T174M (C/T) and Ang rs4762 T174M-T alleles have been linked to IgAV development, as well as being associated with IgAV-induced renal damage (Desong et al., 2010). Familial Mediterranean fever (FMF) is a hereditary auto-inflammatory disease. The causative gene of the FMF is the Mediterranean Fever gene (MEFV). Studies have found that mutations in MEFV are related to the pathogenesis of IgAV, particularly in exon 10 (Ekinci et al., 2019; Yildiz et al., 2020). Gershoni-Baruch et al. (2003) found that 5% of people with FMF also had IgAV, and their detection of point mutations in 52 children with IgAV showed that 10% of children had two mutations that were known to cause FMF. The MEFV gene also encodes the inflammatory protein, pyrin. Pyrin forms a complex network with various inflammatory regulatory factors with its PYRIN domain and the adaptor protein ASC (apoptotic associated speck-like protein) acting as a bridge.

## Epigenetics

Epigenetics is a hereditary modification that affects gene expression and regulation without involving changes in the genomic DNA sequence. It is affected by genetic and external factors and is closely associated with both cell growth and apoptosis. The development of IgAV is influenced by multiple factors such as immunity, infection, and the environment in individuals with genetic susceptibility. There is evidence that epigenetics is involved in IgAV pathogenesis. Luo et al. (2013) found that acetylation and methylation of histone H3 are increased in peripheral blood mononuclear cells from IgAV patients, affecting the IL-4 genetic locus. In addition, there was elevated expression of IL-4, IL-6, IL-13, GATA-3, TIM-1, and CXCL4. These observations suggest that aberrant histone modification may induce EC damage. Abnormal expression of histone-modifying enzymes may affect the histone structure, resulting in modulation of the transcription of specific genes. Besides methylation and histone modification, the roles of non-coding RNAs in IgAV have been extensively studied. The micro-RNAs miR 223-3p and miR146a-5p can activate the NF-κB signaling pathway and participate in vascular inflammation by regulating the expression of IL-6, IL-8, and TNF-α (Koutkia et al., 2001; Brasier, 2010; Nejad et al., 2018). MiR-29b overexpression induced the release of intercellular adhesion molecule-1, IL-1β, IL-6, IL-8, the increase of CyclinA2, CyclinD1, and cell proliferation. It also could inhibit the expressions of TNFAIP3 and NF-kappa-B repressing factor (Cheng et al., 2020). Let-7a not only participates in the regulation of NF-κB signaling and mediates the vascular inflammatory response but is also associated with apoptosis (Iliopoulos et al., 2009; Song et al., 2016). Let-7b and miR148b regulate the expression of N-acetylgalactosamine transferase 2 and β1, 3 galactosamine transferases, key glycosylation enzymes, to influence the normal glycosylation process of IgA1 and regulate the formation of IgA1 immune complexes (Song et al., 2016). However, miR-218-5p reduces EC apoptosis by regulating HMGB1 expression (Yu et al., 2018). Ln RNA NKILA recruits DNMT3A to the CpG island of the KLF4 promoter by mediating NF-κB signaling, promoting methylation and transcriptional inhibition, inhibiting the inflammatory response, and protecting ECs (Zhu et al., 2019). Epigenetics thus modulates the levels of inflammatory factors and the formation of IgA immune complexes, influencing the expression of pathogenetic genes, causing damage to endothelial cells, and aggravating the progression of IgAV.

## Current Situation and Future Perspectives

**FIGURE 2:**
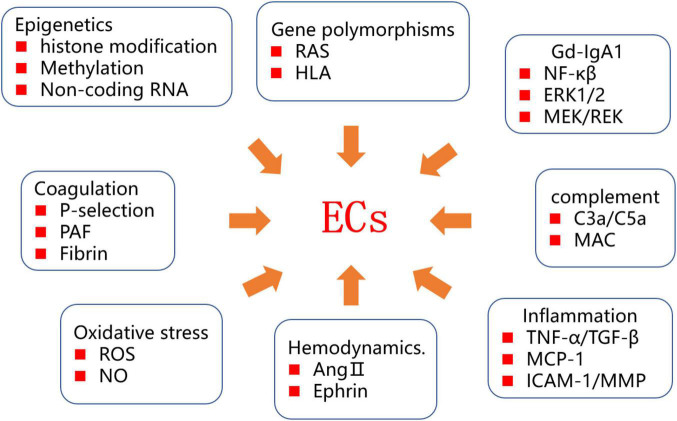
Diagram of IgAV endothelial injury mechanism.

The incidence of IgAV appears to be increasing, possibly in relation to changes in the social environment and food pollution, adversely affecting both the physical and mental health of patients. At present, there has been no thorough investigation of the pathogenesis of IgAV. It is known that ECs are closely linked to the pathogenesis and progression of IgAV. The mechanisms underlying EC injury are complex, and various factors, including IgA1 immune complex deposition, complement system activation, the inflammatory response, and oxidative stress, have been implicated (Figure 2). Recent studies have shown that epigenetics and genetic diversity provide a genetic background for EC injury. ECs continuously express pathogenic genes, eventually leading to changes in the renal extracellular matrix and renal insufficiency.

Clinical studies have found that ACEI and ARB alleviate renal vascular injury in children with IgAVN, reduce urinary protein levels, and delay the progression of the disease. This mechanism may be related to both a reduction in the inflammatory response and the apoptosis of endothelial cells. A recent study by Bian et al. (2011) has found that amlodipine can inhibit Ang II-induced apoptosis of umbilical vein ECs. The precise action of ACEI and ARB on ECs and the specific signaling pathways involved still require verification in large-scale clinical trials.

IgAV requires early diagnosis. Injury to ECs occurs in the early stage of IgAV. Exploring the markers of EC injury and how to apply the detection of EC injury to the early diagnosis of IgAV are the directions of future research. The in-depth study of the relationships between the multiple factors affecting EC injury is necessary for the further understanding of the pathogenesis of IgAV and may also suggest therapeutic targets for the prevention, treatment, and prognosis of IgAV.

## Author Contributions

SX, SH, and YDa participated in the design, execution, and writing of the manuscript. LW and XZ participated in the revision of the manuscript. YDi participated in the design and revision of the manuscript. All authors listed have made a substantial, direct, and intellectual contribution to the work, and approved it for publication.

## Conflict of Interest

The authors declare that the research was conducted in the absence of any commercial or financial relationships that could be construed as a potential conflict of interest.

## Publisher’s Note

All claims expressed in this article are solely those of the authors and do not necessarily represent those of their affiliated organizations, or those of the publisher, the editors and the reviewers. Any product that may be evaluated in this article, or claim that may be made by its manufacturer, is not guaranteed or endorsed by the publisher.
